# Role of smooth muscle cell p53 in pulmonary arterial hypertension

**DOI:** 10.1371/journal.pone.0212889

**Published:** 2019-02-26

**Authors:** Takayuki Wakasugi, Ippei Shimizu, Yohko Yoshida, Yuka Hayashi, Ryutaro Ikegami, Masayoshi Suda, Goro Katsuumi, Masaaki Nakao, Makoto Hoyano, Takeshi Kashimura, Kazufumi Nakamura, Hiroshi Ito, Takashi Nojiri, Tomoyoshi Soga, Tohru Minamino

**Affiliations:** 1 Department of Cardiovascular Biology and Medicine, Niigata University Graduate School of Medical and Dental Sciences, Niigata, Japan; 2 Division of Molecular Aging and Cell Biology, Niigata University Graduate School of Medical and Dental Sciences, Niigata, Japan; 3 Department of Cardiovascular Medicine, Okayama University Graduate School of Medicine, Dentistry and Pharmaceutical Sciences, Okayama, Japan; 4 Department of Biochemistry, National Cerebral and Cardiovascular Center Research Institute, Suita-City, Osaka, Japan; 5 Institute for Advanced Biosciences, Keio University, Yamagata, Japan; Osaka University Graduate School of Medicine, JAPAN

## Abstract

Pulmonary arterial hypertension (PAH) is characterized by remodeling and narrowing of the pulmonary arteries, which lead to elevation of right ventricular pressure, heart failure, and death. Proliferation of pulmonary artery smooth muscle cells (PASMCs) is thought to be central to the pathogenesis of PAH, although the underlying mechanisms are still being explored. The protein p53 is involved in cell cycle coordination, DNA repair, apoptosis, and cellular senescence, but its role in pulmonary hypertension (PH) is not fully known. We developed a mouse model of hypoxia-induced pulmonary hypertension (PH) and found significant reduction of p53 expression in the lungs. Our in vitro experiments with metabolomic analyses and the Seahorse XF extracellular flux analyzer indicated that suppression of p53 expression in PASMCs led to upregulation of glycolysis and downregulation of mitochondrial respiration, suggesting a proliferative phenotype resembling that of cancer cells. It was previously shown that systemic genetic depletion of p53 in a murine PH model led to more severe lung manifestations. Lack of information about the role of cell-specific p53 signaling promoted us to investigate it in our mouse PH model with the inducible Cre-loxP system. We generated a mouse model with SMC-specific gain or loss of p53 function by crossing Myh11-Cre/ERT2 mice with floxed *Mdm4* mice or floxed *Trp53* mice. After these animals were exposed to hypoxia for 4 weeks, we conducted hemodynamic and echocardiographic studies. Surprisingly, the severity of PH was similar in both groups of mice and there were no differences between the genotypes. Our findings in these mice indicate that activation or suppression of p53 signaling in SMCs has a minor role in the pathogenesis of PH and suggest that p53 signaling in other cells (endothelial cells, immune cells, or fibroblasts) may be involved in the progression of this condition.

## Introduction

In patients with pulmonary arterial hypertension (PAH), progressive remodeling and narrowing of the pulmonary arteries eventually result in right ventricular failure and death [1]. Recently, some medications have been shown to improve the clinical outcome [2–4], but the overall prognosis remains unacceptably poor. The mechanisms underlying PAH have not been fully characterized and there is a major unmet medical need in this area. It is well accepted that vascular cells in PAH share similar metabolic features with cancer [5]. Inhibition of mitochondrial respiration, suppression of glucose oxidation, and activation of glycolysis provide advantages for proliferating cancer cells [6]. Pulmonary artery smooth muscle cells (PASMCs), endothelial cells, and fibroblasts also develop a proliferative phenotype by such metabolic alterations, which are thought to promote the pathogenesis of PAH [5]. PASMCs from PAH patients and animal models of PAH display elevation of glycolysis that is associated with reduction of glucose oxidation and mitochondrial respiration [7, 8], resembling the metabolic profile of cancer cells. Vascular cells involved in the pathology of PAH also become resistant to apoptosis, contributing to uncoordinated cell proliferation that leads to intimal and medial thickening [9]. The protein p53 is a well-characterized transcription factor that is involved in cell cycle coordination, DNA repair, apoptosis, and cellular senescence, as well as in maintenance of genomic stability and suppression of tumorigenesis [10]. Activation of p53 signaling inhibits cell proliferation and suppresses glycolysis. Accordingly, it is highly possible that reduced expression of p53 contributes to the pathogenesis of PAH, while activation of p53 signaling may ameliorate PAH. In support of this concept, it was previously reported that systemic p53 deficiency led to exacerbation of hypoxia-induced pulmonary hypertension (PH) in mice [11]. However, there has been no investigation of the role of cell-specific p53 signaling in PH models. We used an inducible Cre-loxP system targeting smooth muscle cells (SMCs) to investigate the influence of cell-specific p53, and demonstrated that SMC-specific gain or loss of p53 function did not lead to exacerbation of hypoxia-induced PH compared with wild-type mice.

## Materials and methods

### Animal models

All animal experiments were conducted in compliance with the protocol reviewed by the Institutional Animal Care and Use Committee of Niigata University and approved by the President of Niigata University. C57BL/6NCr male mice were purchased from SLC Japan (Shizuoka, Japan). Mice carrying floxed Trp53 alleles (*Trp53*^fl/fl^) and Cre recombinase in Myh11-positive cells (Myh11-Cre/ERT2) were purchased from Jackson Laboratories. Then Myh11-Cre/ERT2; *Trp53*^fl/fl^ mice or Myh11-Cre/ERT2; *Mdm4*^fl/fl^ mice were generated by crossing heterozygous Myh11-Cre/ERT2 mice with *Trp53*^fl/fl^ or *Mdm4*^fl/fl^ mice [12]. To induce Cre recombinase, mice received daily injection with 1 mg of tamoxifen (Sigma Life Science T5648-1G) dissolved in 300 μl of corn oil (Sigma Life Science C267-500ML) for 5 days from 7 weeks old. A mouse model of PH was generated by housing the animals in a hypoxic chamber with an atmosphere of 8% O_2_ for 4 weeks from 8 weeks old. Mice were anesthetized with isoflurane for catheter studies. We used a 25G needle with a pressure sensor (FTH-1211B-0018, Primetech) to measure the right ventricle systolic pressure (RVSP), and calculations were done with a LabChart8 (AD Instruments). All studies for pressure measurement are done and analyzed in a blinded way with genotypes. After the animals were euthanized by isoflurane or intraperitoneal barbiturate injection, tissues were quickly corrected for further analyses.

### Cell culture

Human pulmonary arterial smooth muscle cells (PASMCs) and culture medium (CC-3182) were purchased from Lonza, and the cells were incubated according to the manufacturer's instructions. In some experiments, hypoxic stress was introduced by maintaining the cells under a 1% O_2_ atmosphere for 72 hours in a hypoxic chamber (Stemcell Technologies).

### siRNA for p53

Small interfering RNA (si-RNA) targeting p53 (a mixture of si-*TP53* #HSS186391, #HSS186390, and #HSS110905; 10 nM each) and the corresponding negative control (#46–2001) were purchased from Invitrogen. siRNAs were transfected by using Lipofectamine RNAi MAX (Invitrogen, #13778–150) and Opti-MEM (Gibco by Life Technologies, #31985–062). The medium was replaced after 24 hours, and the cells were incubated for a further 24 hours before experiments were performed, unless stated otherwise.

### Echocardiography

Echocardiography was performed with a Vevo 2100 High Resolution Imaging System (Visual Sonics Inc.). To minimize variation of the data, cardiac function was only assessed when the heart rate was within the range of 550–650 /min. All studies for echocardiography were performed and analyzed in a blinded way with genotypes.

### Histological examination

Lung samples were harvested, fixed overnight in 10% formalin, embedded in paraffin, and sectioned for hematoxylin-eosin (HE) staining before examination. The antibodies used were Rabbit Polyclonal Antibody p53 protein (CM5) (Leica NCL-L-p53-CM5p), anti-alpha smooth muscle Actin (abcam, ab21027), and Hoechst (Life Technologies, 33258). Secondary antibody for anti-p53 antibody (CM5) was Donkey Anti-Rabbit IgG H&L (DyLight650)(abcam, ab96894), Donkey Anti-Goat DyLight488 (abcam, ab96931). The concentrations of all primary and secondary antibodies were 1:50 except for Hoechst (1:1000). Stained samples were analyzed with FV1200 confocal microscope (Olympus).

### Western blotting

Whole-cell lysates were prepared in lysis buffer (10 mM Tris-HCl, pH 8, 140 mM NaCl, 5 mM EDTA, 0.025% NaN_3_, 1% Triton X-100, 1% deoxycholate, 0.1% SDS, 1 mM PMSF, 5 μg ml^–1^ leupeptin, 2 μg ml^–1^ aprotinin, 50 mM NaF, and 1 mM Na_2_VO_3_), after which the lysates (40–50 μg) were subjected to SDS-PAGE. Then proteins were transferred to PVDF membranes (Millipore) and incubated with the primary antibody, followed by incubation with horseradish peroxidase-conjugated immunoglobulin G and detection by enhanced chemiluminescence (GE). The primary antibodies for western blotting were anti-p53 antibody (DO-1) (Santa Cruz Biotechnology, sc-126), anti-p53 antibody (Leica, NCL-L-p53-CM5p), and anti-PAN-actin antibody (Cell Signaling Technology, 4968S). All primary antibodies were used at a dilution of 1:1000. The secondary antibody for anti-p53 antibody (sc-126) was peroxidase-conjugated AffiniPure goat anti-mouse IgG(H+L) (Jackson Immunoresearch, 115-035-003), while peroxidase-conjugated AffiniPure goat anti-rabbit IgG (H+L) (Jackson Immunoresearch, 111-035-003) was used for anti-p53 antibody (NCL-L-p53-CM5p) and anti-PAN-actin antibody (4968S).

### Extracellular flux assay

Cellular oxygen consumption and extracellular acidification were measured with a Seahorse XF extracellular flux analyzer according to the manufacturer’s instructions (Agilent). PASMCs were cultured in a Seahorse XF 24-well assay plate in antibiotic-free medium at a density of 20,000 cells/well, and were treated with siRNA for p53 (si-*TP53*; Invitrogen) and negative control siRNA (Invitrogen) after attachment. The medium was replaced with antibiotic-containing medium after 24 hours. After incubation for a further 24 hours, the plates were washed and the medium was replaced with pre-warmed running medium (XF base medium supplemented with 10% D-glucose, 100 mM pyruvate, and 200 mM glutamine for the mitochondrial stress test, or with 200 mM glutamine alone for the glycolysis stress test). Then incubation was performed in a non-CO_2_ incubator for 60 min at 37°C. The basal oxygen consumption rate and extracellular acidification rate were recorded for 24 min, followed by performance of the mitochondrial stress test (1μM oligomycin, 2 μM FCCP, and 0.5 μM rotenone/antimycin A) and glycolysis stress test (10 mM glucose, 1 μM oligomycin, and 50 mM 2-DG). All reagents were obtained from the Seahorse XF Cell Mito Stress Test Kit (Seahorse Bioscience, #103015–100) and the Seahorse XF Glycolysis Stress Test Kit (Seahorse Bioscience, #103020–100).

### Metabolomic analyses

Metabolomic analyses were done according to the method of Soga et al. by using capillary electrophoresis/mass spectrometry (CE/MS), as described previously [13]. Briefly, 1,000,000 PASMCs in a 6-cm dish were treated with si-RNA as described above, washed twice with ice cold 5% mannitol, and let stand for 10 min at room temperature in methanol containing L-methionine sulfone (25 μM, Alfa Aesar A17027), MES (25 μM, Dojindo 341–01622), and CSA (25 μM, Wako 037–01032). Then the cells were harvested with a cell scraper and 400 μL of supernatant was collected after vortexing for 30 seconds. After addition of CHCl_3_ (400 μl) and distilled water (200 μl) with thorough mixing, centrifugation was performed at 10,000 g for 3 minutes at 4°C. Next, the aqueous layer (400 μl) was transferred to an ultrafiltration tube (UltrafreeMC-PLHCC, Human Metabolome Technologies, UFC3LCCNB-HMT), followed by centrifugation at 9,100 g for 3 hours at 4°C. Finally, 320 μL of the filtrate was sent to the Institute for Advanced Biosciences at Keio University for further analyses.

### Statistical analysis

Results are shown as the mean ± SEM. Outliers and abnormal values were excluded by boxplot analyses. Differences between groups were examined by the two-tailed Student’s *t*-test or two-way ANOVA, which were followed by Tukey’s multiple comparison test for comparisons among more than two groups. In all analyses, *P*<0.05 was considered statistically significant. Analyses were done with SPSS software (version 24).

## Results

### Generation of the PH model with hypoxic stress

**Fig 1:**
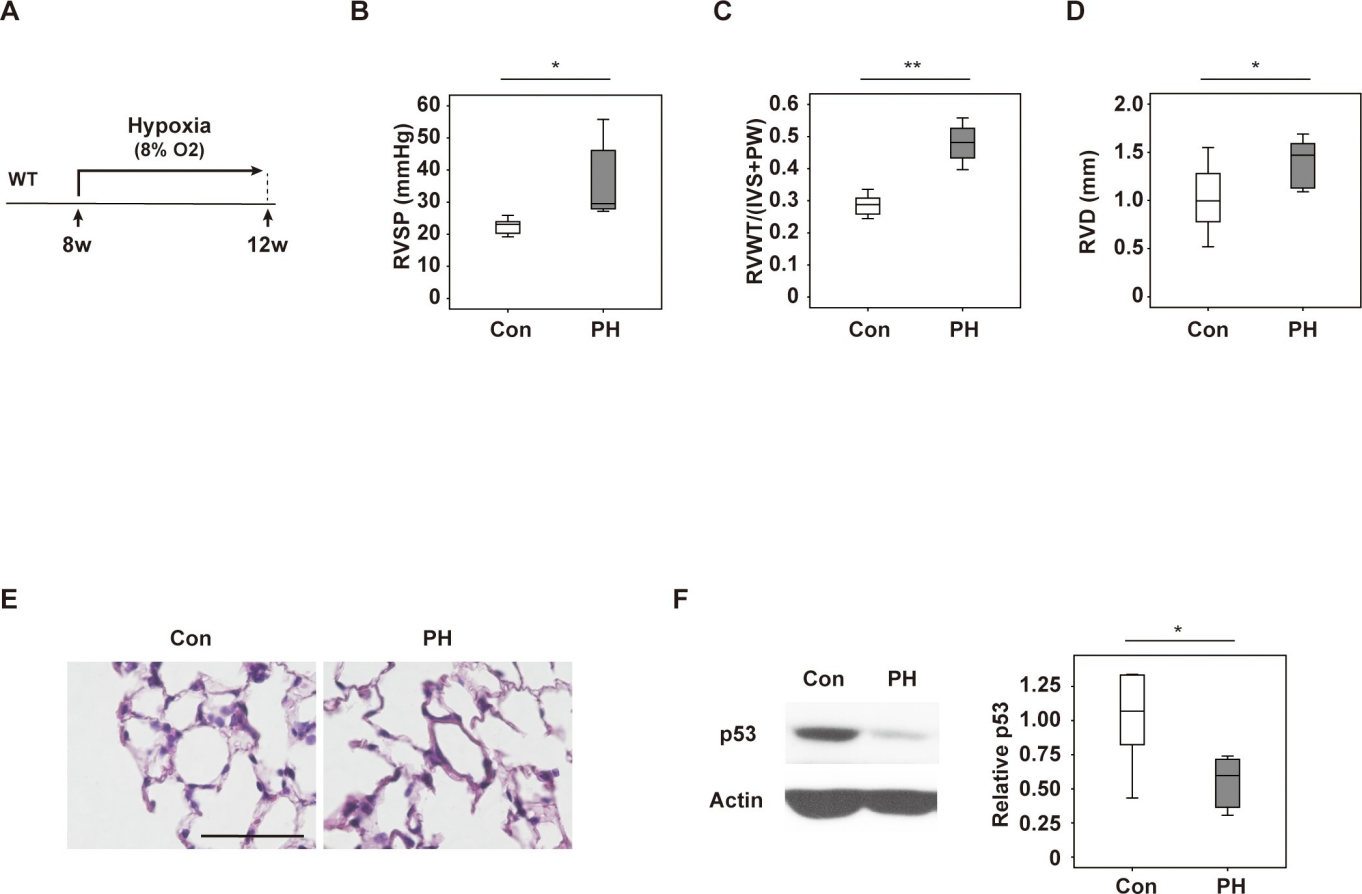
Generation of a mouse model of hypoxia-induced PH. (A) Scheme for generating the mouse model of hypoxia-induced PH. Eight-week-old mice were housed in a hypoxic chamber (8% O_2_) for 4 weeks before experiments were performed. (B) Right ventricular systolic pressure (RVSP) in mice exposed to normoxia (Con) or hypoxia (PH) (n = 9, 8). (C, D) Right ventricular wall thickness (RVWT) (n = 8, 10) (C) and right ventricular diameter (RVD) (n = 8, 10) (D) in the indicated mice. (E) Hematoxylin and eosin (HE) staining of tissues harvested from the indicated mice. Scale bar = 50μm. (F) Western blot analysis of p53 in lungs from the indicated mice. The right panel displays quantification of p53 relative to the actin loading control (n = 5, 5). Data are shown as the mean ± s.e.m. *P<0.05, **P<0.01 by the 2-tailed Student’s *t*-test (B-D, F).

We generated a murine PH model by exposing C57BL6/NCr mice to hypoxia (8% O_2_) for 4 weeks (Fig 1A). Exposure to hypoxia led to a significant increase of right ventricular systolic pressure (RVSP) (Fig 1B), right ventricular wall thickness (RVWT) (Fig 1C), and right ventricular diameter (RVD) (Fig 1D). In contrast, left ventricular systolic function (S1A Fig), left ventricular end-systolic diameter (S1B Fig), and heart rate (S1C Fig) were comparable between normoxic mice and mice with hypoxia-induced PH. Pathological examination showed pulmonary artery wall thickening in our PH model (Fig 1E), indicating that it displayed features of pulmonary arterial hypertension (PAH). Because p53 null mice were reported to show advanced changes of PH [11], we investigated the overall level of p53 expression in PH lungs and found significant reduction of p53 (Fig 1F), which might contribute to vascular remodeling with progression of PH.

### Suppression of p53 expression in PASMCs upregulates glycolysis

**Fig 2:**
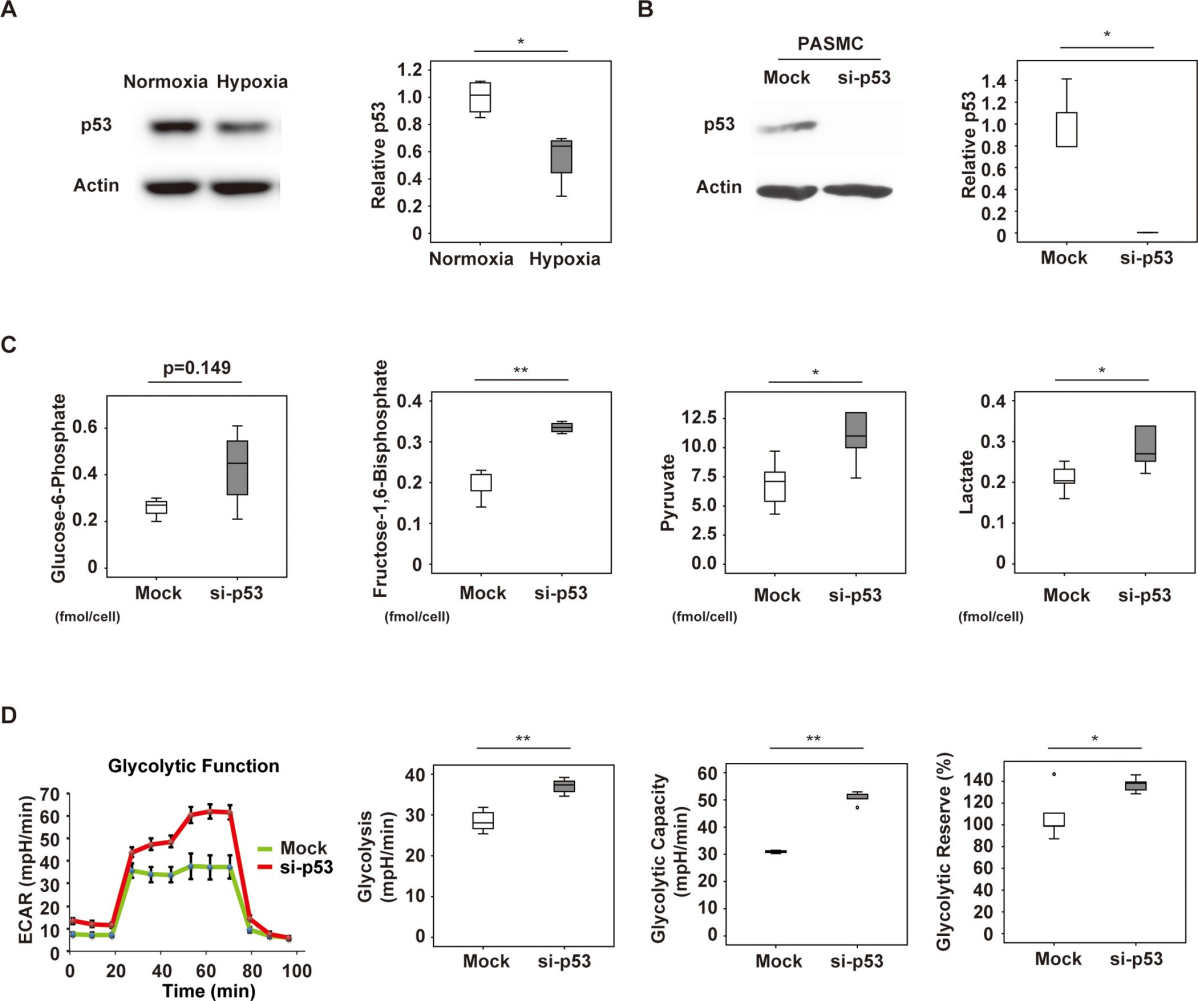
Glycolysis in p53-depleted pulmonary arterial smooth muscle cells (PASMCs). (A) Western blot analysis of p53 in PASMCs exposed to normoxia (Con) or hypoxia (Hypoxia). The right panel shows quantification of p53 relative to the actin loading control (n = 4,4). (B) Western blot analysis of PASMCs treated with siRNA for p53 (si-p53) or control siRNA (Mock). The right panel displays quantification of p53 relative to the actin loading control (n = 3,3). (C) Glucose-6-phosphate (n = 3,4), fructose-1,6-bisphosphate (n = 5,4), pyruvate (n = 5,5), and lactate (n = 5,5) levels determined by metabolomic analyses in PASMCs treated with siRNA for p53 (si-p53) or control siRNA (Mock). (D) PASMCs were treated with siRNA for p53 (si-p53) or control siRNA (Mock), followed by Seahorse XF extracellular flux analysis of glycolytic function, including the extracellular acidification rate (ECAR), glycolysis (n = 5,5), glycolytic capacity (n = 3,5), and glycolytic reserve (n = 5,5). Results represent the mean ± s.e.m. *P<0.05, **P<0.01 by the 2-tailed Student’s *t*-test (A–D). In Fig 2C, PASMCs treated with siRNA for p53 (si-p53) or control siRNA (Mock) were analyzed in 5 groups each, but glucose-6-phosphate became undetectable in 2 Mock groups and 1 si-p53 group. Boxplot analysis of fructose-1,6-bisphosphate data identified 1 abnormal value in the si-p53 group, and this was excluded from the figure and from analysis. In Fig 2D, glycolytic function was analyzed in 5 groups each of PASMCs treated with siRNA for p53 (si-p53) or control-siRNA (Mock), but boxplot analysis identified 2 abnormal values for glycolytic capacity in the Mock group and these were excluded from analysis.

Because p53 expression was reduced in PH lungs, we performed in vitro investigation of the role of p53 in PASMCs. We found that exposure to hypoxic stress led to reduction of p53 expression by these cells (Fig 2A). To further characterize the role of p53 in PASMCs, we used siRNA targeting p53 to suppress its expression (Fig 2B). Metabolomic studies showed that suppression of p53 led to a marked increase of glucose-6-phosphate in these cells, as well as a significant increase of fructose-1,6-bisphosphate, pyruvate, and lactate (Fig 2C). Evaluation of glycolysis by using the Seahorse XF extracellular flux analyzer showed that suppression of p53 expression increased glycolysis, glycolytic capacity, and the glycolytic reserve (Fig 2D). These results indicated that inhibition of p53 led to a metabolic shift toward glycolysis in PASMCs.

### Suppression of p53 inhibits the TCA cycle and mitochondrial respiration in PASMCs

**Fig 3:**
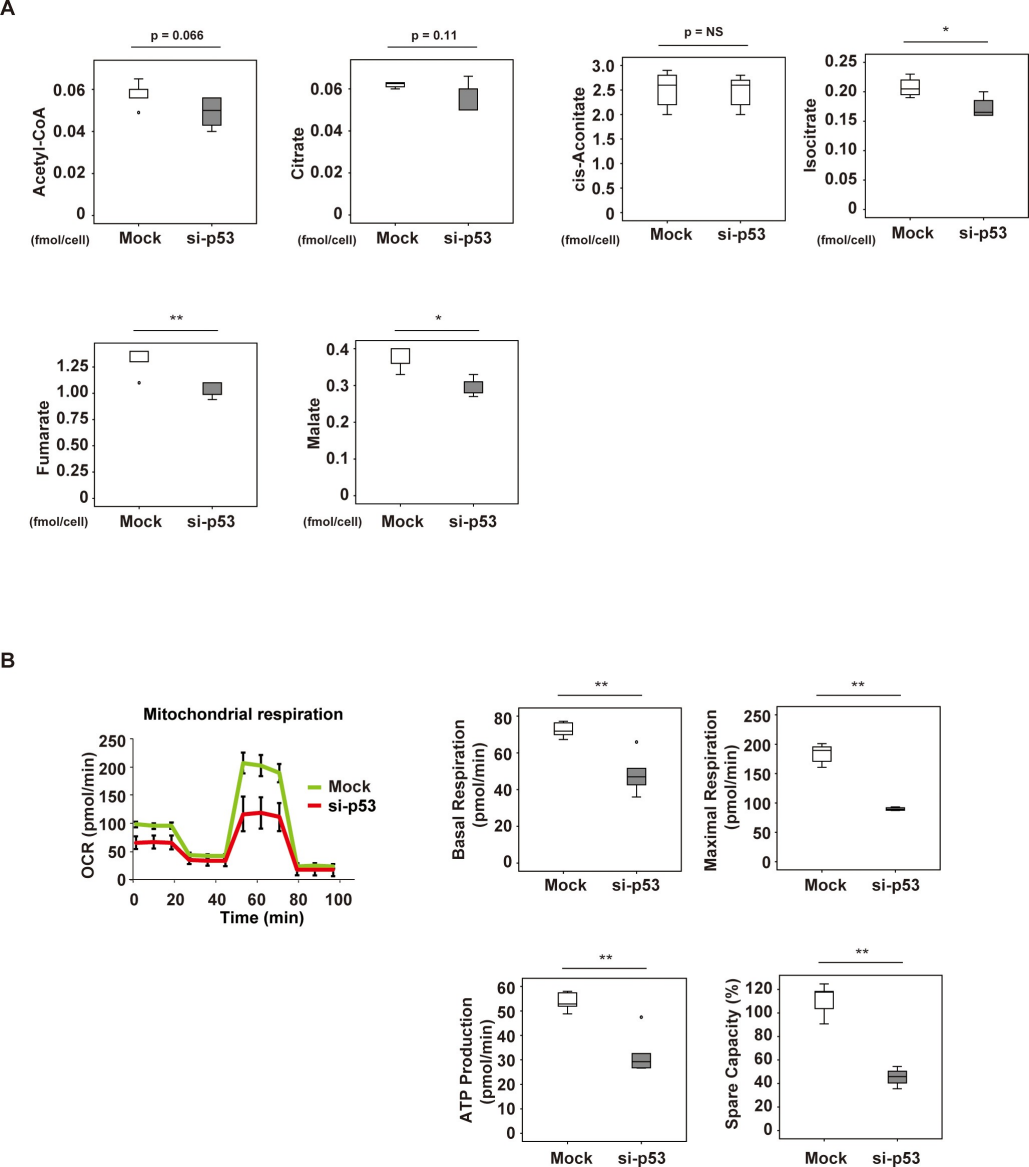
Mitochondrial respiration in p53-depleted pulmonary arterial smooth muscle cells (PASMCs). (A) Levels of acetyl-CoA (n = 5,5), citrate (n = 4,5), cis-aconitate (n = 5,5), isocitrate (n = 4,4), fumarate (n = 5,5) and malate (n = 5,5) determined by metabolomic analysis in PASMCs treated with siRNA for p53 (si-p53) or control siRNA (Mock). (B) PASMCs were treated with siRNA for p53 (si-p53) or control siRNA (Mock), followed by Seahorse XF extracellular flux analysis of mitochondrial respiration, including the oxygen consumption rate (OCR), basal respiration (n = 5,5), maximal respiration (n = 5,4), ATP production (n = 5,4) and spare capacity (n = 5,4). Results represent the mean ± s.e.m. *P<0.05, **P<0.01 by the 2-tailed Student’s *t*-test (A, B). In Fig 3A, PASMCs treated with siRNA for p53 (si-p53) or control siRNA (Mock) were analyzed in 5 groups each. However, boxplot analysis identified 1 abnormal value for citrate in the Mock group, 1 for isocitrate in the Mock group, and 1 for isocitrate in the si-p53 group, and these values were excluded from analyses. In Fig 3B, mitochondrial respiration was analyzed in 5 groups each of PASMCs treated with siRNA for p53 (si-p53) or control siRNA (Mock). Boxplot analysis identified 1 abnormal value each for maximal respiration, ATP production, and Spare capacity in the si-p53 group, and these values were excluded from analyses.

Next, we tried to assess the changes of the TCA cycle and mitochondrial respiration with p53 inhibition in PASMCs. Metabolomic studies indicated that acetyl-CoA and citrate were reduced by suppression of p53 expression. There was no change of cis-aconitate, but isocitrate, fumarate, and malate levels were significantly reduced by p53 suppression (Fig 3A). Next, we evaluated mitochondrial respiration by using the Seahorse extracellular flux analyzer, revealing that p53 inhibition reduced basal respiration, maximal respiration, ATP production, and spare capacity (Fig 3B). These results indicated that both the TCA cycle and mitochondrial respiration were suppressed in PASMCs by inhibition of p53. Taken together, the in vitro data indicated that suppression of p53 expression in PASMCs led to metabolic remodeling, which was characterized by activation of glycolysis and inhibition of mitochondrial respiration, mimicking the features of cancer cell metabolism.

### Smooth muscle cell-specific gain or loss of p53 function has no impact on hypoxia-induced PH

**Fig 4:**
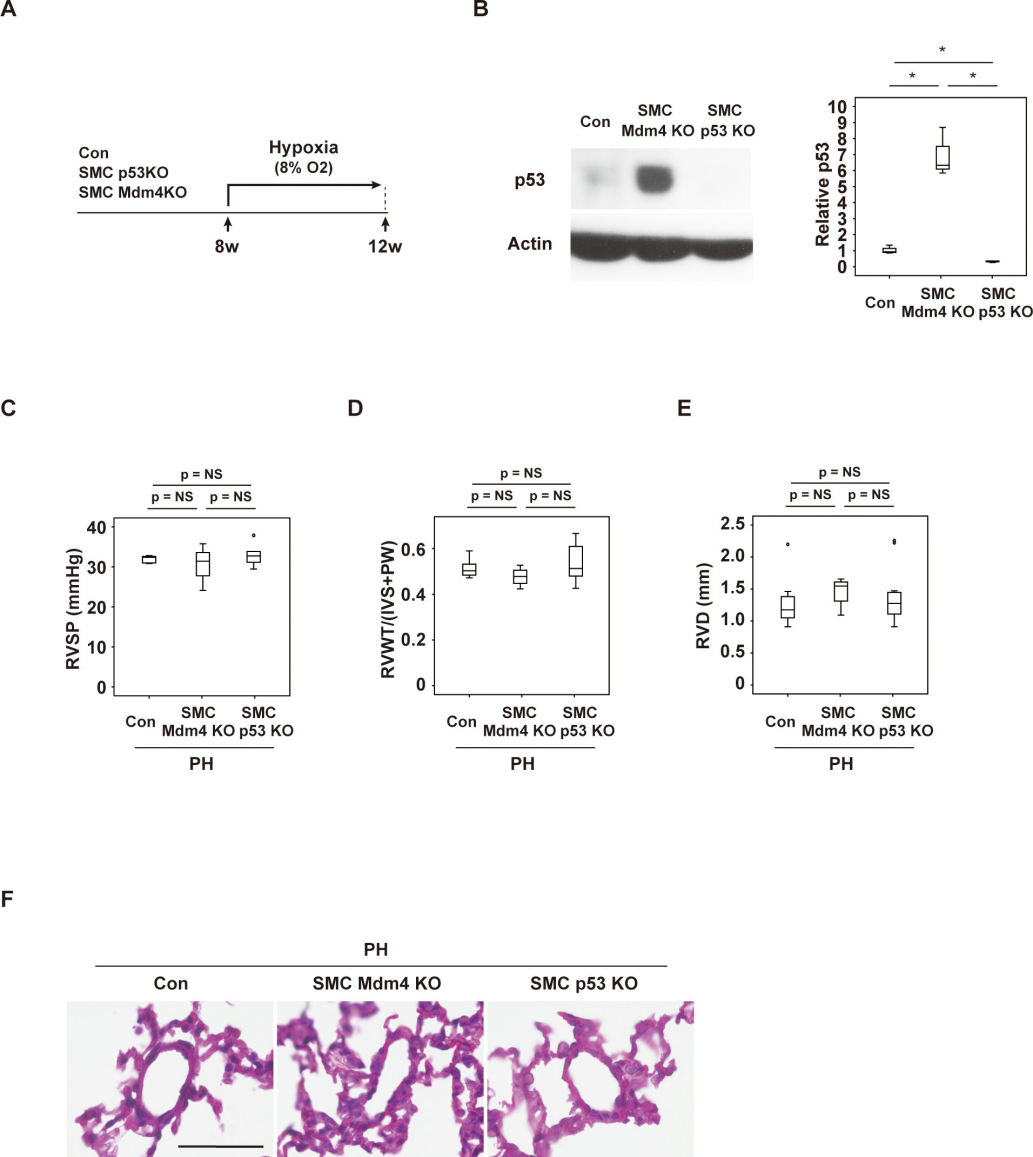
Mouse models of PH with SMC-specific gain or loss of p53 function. Scheme for generation of mice with hypoxia-induced PH. (B) Western blot analysis of p53 expression in mice with SMC-specific gain of p53 function (Myh11-Cre/ERT2; *Mdm4*^fl/fl^ (SMC-Mdm4KO)) or loss of p53 function (Myh11-Cre/ERT2; *Trp53*^fl/fl^ (SMC-p53KO)). The right panel shows quantification of p53 relative to the actin loading control in the indicated groups (n = 3,3,3). (C) Right ventricular systolic pressure (RVSP) of the indicated mice housed under normoxia (Con) or hypoxia (PH) (n = 9,4,11). (D, E) Right ventricular wall thickness (RVWT) (n = 9,4,11) (D) and right ventricular diameter (RVD) (n = 9,4,11) (E) of the indicated mice housed under hypoxia (PH). (F) Hematoxylin and eosin (HE) staining of tissues from the indicated mice. Scale bar = 50 μm. Data represent the mean ± s.e.m. *P<0.05, **P<0.01 by the 2-tailed Student’s *t*-test (B), or 2-way ANOVA followed by Tukey’s multiple comparison test (C–E).

To further investigate the role of p53 signaling in PASMCs, we generated mouse models with SMC-specific depletion or activation of p53. The SMC-specific p53 depletion model was generated by crossing Myh11-Cre/ERT2 mice with floxed p53 mice (Myh11-Cre/ERT2; *Trp53*^fl/fl^ (SMC-p53KO)), while the SMC-specific p53 activation model was generated by crossing Myh11-Cre/ERT2 mice with *Mdm4*^fl/fl^ mice (Myh11-Cre/ERT2; *Mdm4*^fl/fl^ (SMC-Mdm4KO)). Myh11-Cre/ERT2 mice were analyzed as controls. Myh11-Cre/ERT2 (control), SMC-p53KO mice, and SMC-Mdm4KO mice were housed under hypoxic conditions (8% O_2_) for 4 weeks (Fig 4A). First, we investigated p53 expression in aortic tissue with abundant SMCs, revealing that p53 expression was increased in SMC-Mdm4 KO mice and reduced in SMC-p53KO mice (Fig 4B). We also confirmed an increase of p53-positive SMC number in pulmonary arteries of SMC-Mdm4KO mice, while SMC-p53KO mice showed a significant reduction of p53-positive SMC (S2A Fig). Surprisingly, hemodynamic studies showed that RVSP was comparable among the genotypes (Fig 4C). Likewise, echocardiography indicated that RVWT and RVD were similar among the control mice, SMC p53KO mice, and SMC Mdm4KO mice (Fig 4D and 4E). Left ventricular systolic function (S2B Fig), left ventricular end-systolic diameter (S2C Fig), and heart rate (S2D Fig) were also comparable among the genotypes. Furthermore, there was no difference in the severity of pulmonary artery narrowing among the genotypes (Fig 4F). These results indicated that vascular smooth muscle cell-specific gain or loss of p53 function had no effect on hypoxia-induced PH.

## Discussion

Because p53 regulates the cell cycle, DNA repair, apoptosis, cellular senescence, and cellular metabolism, it has an important role in the maintenance of homeostasis [10]. We previously reported that p53-induced cellular senescence is a critical process in cardiovascular and metabolic diseases [14–19]. We found that p53 signaling was upregulated in cardiomyocytes, vascular cells, adipocytes, and immune cells in cardiovascular and metabolic diseases, whereas p53 inactivation alleviated these age-related disorders. In cardiovascular and metabolic diseases, senescent cells are characterized by growth arrest and alterations of the genetic profile that contribute to initiation and maintenance of chronic sterile inflammation associated with tissue remodeling [20]. Accordingly, suppression of cellular senescence or elimination of senescent cells may have the potential to become next generation therapy for such age-related disorders [21–23].

PAH develops due to remodeling and narrowing of the pulmonary arteries. In this condition, vascular cells such as PASMCs, endothelial cells, and fibroblasts exhibit a proliferative phenotype, and uncoordinated proliferation of these cells contributes to vascular remodeling. In PAH, pulmonary vascular cells develop an anti-apoptotic phenotype and undergo metabolic remodeling that is characterized by increased glycolysis, reduced glucose oxidation, and reduced mitochondrial respiration [5]. This is similar to the metabolic profile of cancer cells, which is recognized to provide a survival advantage for proliferating cells. Since p53 signaling inhibits cell proliferation and also suppresses glycolysis, we hypothesized that downregulation of p53 could exacerbate the pathology of PAH, whereas p53 activation may ameliorate PAH. In support of this concept, it was previously reported that systemic p53 deficiency promoted hypoxia-induced PH in mice [11]. Accordingly, we attempted to characterize p53 signaling in specific cell types. PASMCs from PAH patients are reported to show increased glycolysis, reduced glucose oxidation, and reduced mitochondrial respiration, with these metabolic changes having a critical role in vascular remodeling [9, 24]. Therefore, we focused on p53 signaling in PASMCs and its potential role in PH. As expected, our in vitro studies showed that genetic suppression of p53 in PASMCs led to upregulation of glycolysis and downregulation of mitochondrial respiration. The metabolic shift was similar to that seen in cancer cells, and we predicted it would promote the progression of PH in an animal model. However, we found that SMC-specific gain or loss of p53 function had no phenotypic impact in a mouse model of hypoxia-induced PH. Hemodynamic studies and echocardiography showed that RVSP, RVWT, and RVD were comparable among control mice, SMC-p53KO mice, and SMC-Mdm4KO mice exposed to hypoxia. These findings indicated that activation or suppression of p53 signaling in SMCs has a minor role in the pathology of PH and suggest that p53 signaling in other cells (including endothelial cells, immune cells, or fibroblasts) may be involved in progression of this condition. PH is recognized to be a multiorgan disease, and tissue remodeling is known to occur in various organs, including the skeletal muscle, spleen, and bone marrow [5, 9]. Thus, activation of p53 in SMCs alone may not have been sufficient to influence the course of PH, which would be mediated by coordinated modulation of p53 signaling in other cells.

A potential limitation of this study is the model analyzed. A hypoxia-induced PH model is widely used for PH research; however, it is known that pathological changes are not completely compatible with the pathology of human PAH lung. For example, plexiform lesion does not develop in most cases, and therefore fundamental questions that remain to be answered are whether mouse models are suitable for understanding pathologies in PH patients. Another potential limitation of our study is related to divergent functions of p53. It is well known that p53 is involved in a broad spectrum of cellular processes including cell cycle, apoptosis and cellular senescence in addition to its role in maintaining cell metabolism. Although previous studies suggest that p53 may control pulmonary SMC proliferation via regulation of metabolism, p53 may affect SMC proliferation and/or survival via other mechanisms. Further studies would be required to understand the role of p53 in the pathogenesis of PH.

## Supporting information

S1 Fig Echocardiographic findings in the mouse PH model. (A–C) Echocardiographic findings of murine PH model. (A) Fractional shortening (FS) (n = 10,10), (B) left ventricular systolic dimension (LVDs) (n = 10,10), and (C) heart rate (n = 12,11). Data represent the mean ± s.e.m. *P<0.05, **P<0.01 by the 2-tailed Student’s *t*-test (A–C).
(DOCX)

S2 Fig Echocardiographic findings in PH mice with SMC-specific gain or loss of p53 function. (A) Quantitative data for immunofluorescence study analyzing the number of p53-positive vascular smooth muscle cells (VSMCs) in SMC-specific gain of p53 function (Myh11-Cre/ERT2; *Mdm4*^fl/fl^ (SMC-Mdm4KO)) or loss of p53 function (Myh11-Cre/ERT2; *Trp53*^fl/fl^ (SMC-p53KO)) models (n = 4,4,4). (B–D) Echocardiographic findings in PH mice with SMC-specific gain of p53 function (Myh11-Cre/ERT2; *Mdm4*^fl/fl^ (SMC-Mdm4KO)) or loss of p53 function (Myh11-Cre/ERT2; *Trp53*^fl/fl^ (SMC-p53KO)). (B) Fractional shortening (FS) (n = 9,4,11), (C) left ventricular systolic dimension (LVDs) (n = 9,4,11), and (D) heart rate (n = 9,4,11) in the indicated mice. Data represent the mean ± s.e.m. Analyses were done by 2-way ANOVA, followed by Tukey’s multiple comparison test (A–D).
(DOCX)

## References

[1] Archer SL, Weir EK, Wilkins MR. Basic science of pulmonary arterial hypertension for clinicians: new concepts and experimental therapies. Circulation. 2010;121(18):2045–66. 10.1161/CIRCULATIONAHA.108.847707 20458021 PMC2869481

[2] Ghofrani HA, Galie N, Grimminger F, Grunig E, Humbert M, Jing ZC, et al Riociguat for the treatment of pulmonary arterial hypertension. N Engl J Med. 2013;369(4):330–40. 10.1056/NEJMoa1209655 . 23883378

[3] Galie N, Barbera JA, Frost AE, Ghofrani HA, Hoeper MM, McLaughlin VV, et al Initial Use of Ambrisentan plus Tadalafil in Pulmonary Arterial Hypertension. New England Journal of Medicine. 2015;373(9):834–44. 10.1056/NEJMoa1413687 WOS:000360171700009. 26308684

[4] Sitbon O, Channick RN, Delcroix M, Ghofrani HA, Jansa P, Le Brun FO, et al Macitentan reduces the risk of morbidity and mortality irrespective of the presence or absence of right ventricular (RV) impairment: Results from the randomised SERAPHIN trial in pulmonary arterial hypertension (PAH). European Respiratory Journal. 2014;44. WOS:000209782505205.

[5] Paulin R, Michelakis ED. The metabolic theory of pulmonary arterial hypertension. Circ Res. 2014;115(1):148–64. 10.1161/CIRCRESAHA.115.301130 . 24951764

[6] Heiden MGV, Cantley LC, Thompson CB. Understanding the Warburg Effect: The Metabolic Requirements of Cell Proliferation. Science. 2009;324(5930):1029–33. 10.1126/science.1160809 WOS:000266246700031. 19460998 PMC2849637

[7] Boehme J, Sun XT, Tormos KV, Gong WH, Kellner M, Datar SA, et al Pulmonary artery smooth muscle cell hyperproliferation and metabolic shift triggered by pulmonary overcirculation. American Journal of Physiology-Heart and Circulatory Physiology. 2016;311(4):H944–H57. 10.1152/ajpheart.00040.2016 WOS:000390116100008. 27591215 PMC5114466

[8] Xiao YB, Peng HY, Hong CL, Chen Z, Deng XC, Wang AP, et al PDGF Promotes the Warburg Effect in Pulmonary Arterial Smooth Muscle Cells via Activation of the PI3K/AKT/mTOR/HIF-1 alpha Signaling Pathway. Cellular Physiology and Biochemistry. 2017;42(4):1603–13. 10.1159/000479401 WOS:000412247700027. 28738389

[9] Sutendra G, Michelakis ED. The metabolic basis of pulmonary arterial hypertension. Cell Metab. 2014;19(4):558–73. 10.1016/j.cmet.2014.01.004 . 24508506

[10] Lane DP. Cancer—P53, Guardian of the Genome. Nature. 1992;358(6381):15–6. 10.1038/358015a0 WOS:A1992JB34100026. 1614522

[11] Mizuno S, Bogaard HJ, Kraskauskas D, Alhussaini A, Gomez-Arroyo J, Voelkel NF, et al p53 Gene deficiency promotes hypoxia-induced pulmonary hypertension and vascular remodeling in mice. American Journal of Physiology-Lung Cellular and Molecular Physiology. 2011;300(5):L753–L61. 10.1152/ajplung.00286.2010 WOS:000290088800009. 21335523

[12] Grier JD, Xiong S, Elizondo-Fraire AC, Parant JM, Lozano G. Tissue-specific differences of p53 inhibition by Mdm2 and Mdm4. Mol Cell Biol. 2006;26(1):192–8. 10.1128/MCB.26.1.192-198.2006 16354690 PMC1317622

[13] Hirayama A, Kami K, Sugimoto M, Sugawara M, Toki N, Onozuka H, et al Quantitative metabolome profiling of colon and stomach cancer microenvironment by capillary electrophoresis time-of-flight mass spectrometry. Cancer Res. 2009;69(11):4918–25. 10.1158/0008-5472.CAN-08-4806 . 19458066

[14] Sano M, Minamino T, Toko H, Miyauchi H, Orimo M, Qin Y, et al p53-induced inhibition of Hif-1 causes cardiac dysfunction during pressure overload. Nature. 2007;446(7134):444–8. 10.1038/nature05602 . 17334357

[15] Shimizu I, Yoshida Y, Katsuno T, Tateno K, Okada S, Moriya J, et al p53-Induced Adipose Tissue Inflammation Is Critically Involved in the Development of Insulin Resistance in Heart Failure (vol 15, pg 51, 2012). Cell Metabolism. 2012;15(5):787–. 10.1016/j.cmet.2012.04.014 WOS:000303694100025. 22225876

[16] Shimizu I, Yoshida Y, Moriya J, Nojima A, Uemura A, Kobayashi Y, et al Semaphorin3E-induced inflammation contributes to insulin resistance in dietary obesity. Cell Metab. 2013;18(4):491–504. 10.1016/j.cmet.2013.09.001 . 24093674

[17] Shimizu I, Yoshida Y, Suda M, Minamino T. DNA damage response and metabolic disease. Cell Metab. 2014;20(6):967–77. 10.1016/j.cmet.2014.10.008 . 25456739

[18] Yokoyama M, Okada S, Nakagomi A, Moriya J, Shimizu I, Nojima A, et al Inhibition of Endothelial p53 Improves Metabolic Abnormalities Related to Dietary Obesity. Cell Reports. 2014;7(5):1691–703. 10.1016/j.celrep.2014.04.046 WOS:000338324200032. 24857662

[19] Yoshida Y, Shimizu I, Katsuumi G, Jiao S, Suda M, Hayashi Y, et al p53-Induced inflammation exacerbates cardiac dysfunction during pressure overload. J Mol Cell Cardiol. 2015;85:183–98. 10.1016/j.yjmcc.2015.06.001 . 26055447

[20] Tchkonia T, Zhu Y, van Deursen J, Campisi J, Kirkland JL. Cellular senescence and the senescent secretory phenotype: therapeutic opportunities. J Clin Invest. 2013;123(3):966–72. 10.1172/JCI64098 23454759 PMC3582125

[21] Chang JH, Wang YY, Shao LJ, Laberge RM, Demaria M, Campisi J, et al Clearance of senescent cells by ABT263 rejuvenates aged hematopoietic stem cells in mice. Nature Medicine. 2016;22(1):78–+. 10.1038/nm.4010 WOS:000367590700018. 26657143 PMC4762215

[22] Baar MP, Brandt RM, Putavet DA, Klein JD, Derks KW, Bourgeois BR, et al Targeted Apoptosis of Senescent Cells Restores Tissue Homeostasis in Response to Chemotoxicity and Aging. Cell. 2017;169(1):132–47 e16. 10.1016/j.cell.2017.02.031 28340339 PMC5556182

[23] Roos CM, Zhang B, Palmer AK, Ogrodnik MB, Pirtskhalava T, Thalji NM, et al Chronic senolytic treatment alleviates established vasomotor dysfunction in aged or atherosclerotic mice. Aging Cell. 2016;15(5):973–7. Epub 2016/02/13. 10.1111/acel.12458 26864908 PMC5013022

[24] Thenappan T, Ormiston ML, Ryan JJ, Archer SL. Pulmonary arterial hypertension: pathogenesis and clinical management. BMJ. 2018;360:j5492 Epub 2018/03/16. 10.1136/bmj.j5492 . 29540357 PMC6889979

