# Supplementary material for: Role of smooth muscle cell p53 in pulmonary arterial hypertension
Source: PLoS One. 2019 Feb 26;14(2):e0212889. doi: 10.1371/journal.pone.0212889 (PMC6391010; doi:10.1371/journal.pone.0212889)
Supplement: S1 Fig — (A–C) Echocardiographic findings of murine PH model. (A) Fractional shortening (FS) (n = 10,10), (B) left ventricular systolic dimension (LVDs) (n = 10,10), and (C) heart rate (n = 12,11). Data represent the mean ± s.e.m. *P<0.05, **P<0.01 by the 2-tailed Student’s t-test (A–C). (DOCX) [file pone.0212889.s001.docx]

**
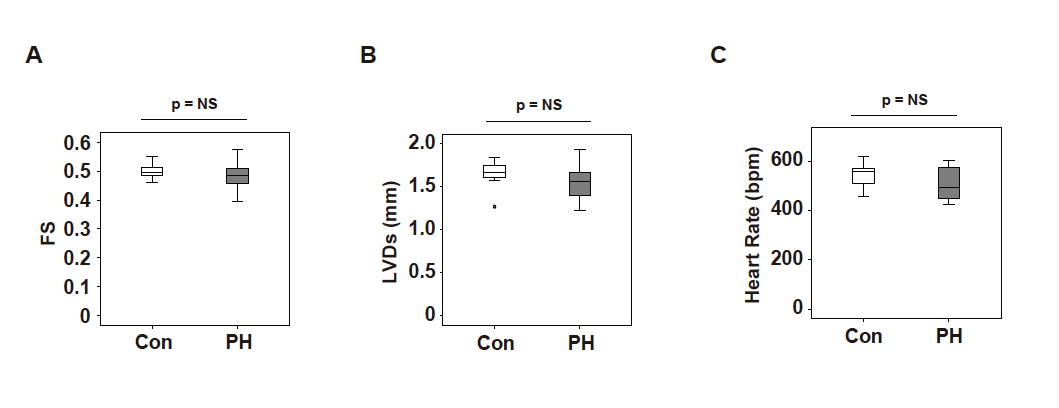
**

**S1 Fig. Echocardiographic findings in the mouse PH model**

(A–C) Echocardiographic findings of murine PH model. (A) Fractional shortening (FS) (n=10,10), (B) left ventricular systolic dimension (LVDs) (n=10,10), and (C) heart rate (n=12,11). Data represent the mean ± s.e.m. **P*<0.05, ***P*<0.01 by the 2-tailed Student’s *t*-test (A–C).
