# Supplementary material for: Role of smooth muscle cell p53 in pulmonary arterial hypertension
Source: PLoS One. 2019 Feb 26;14(2):e0212889. doi: 10.1371/journal.pone.0212889 (PMC6391010; doi:10.1371/journal.pone.0212889)
Supplement: S2 Fig — (A) Quantitative data for immunofluorescence study analyzing the number of p53-positive vascular smooth muscle cells (VSMCs) in SMC-specific gain of p53 function (Myh11-Cre/ERT2; Mdm4fl/fl (SMC-Mdm4KO)) or loss of p53 function (Myh11-Cre/ERT2; Trp53fl/fl (SMC-p53KO)) models (n = 4,4,4). (B–D) Echocardiographic findings in PH mice with SMC-specific gain of p53 function (Myh11-Cre/ERT2; Mdm4fl/fl (SMC-Mdm4KO)) or loss of p53 function (Myh11-Cre/ERT2; Trp53fl/fl (SMC-p53KO)). (B) Fractional shortening (FS) (n = 9,4,11), (C) left ventricular systolic dimension (LVDs) (n = 9,4,11), and (D) heart rate (n = 9,4,11) in the indicated mice. Data represent the mean ± s.e.m. Analyses were done by 2-way ANOVA, followed by Tukey’s multiple comparison test (A–D). (DOCX) [file pone.0212889.s002.docx]

**
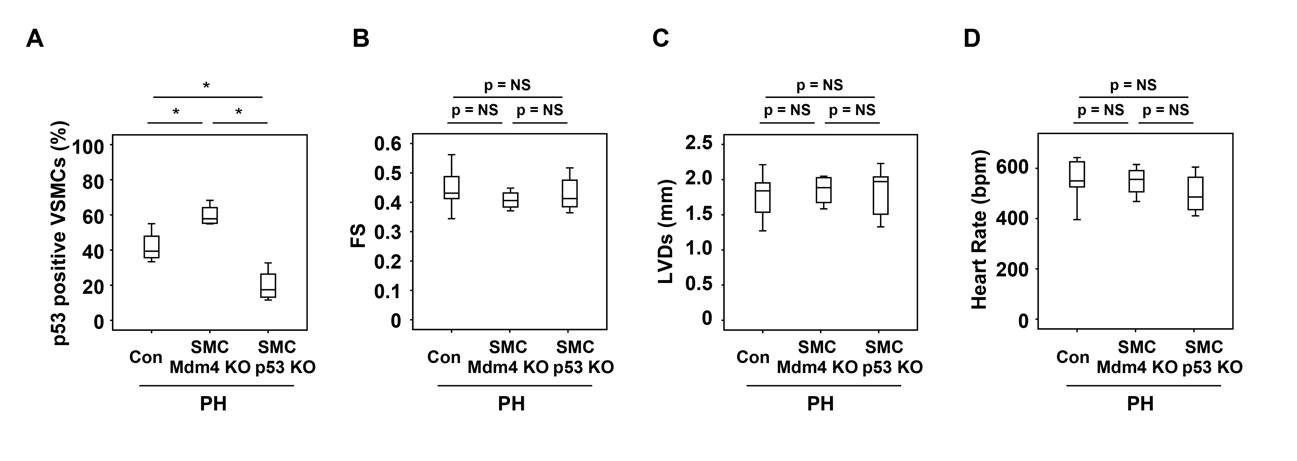
**

**S2 Fig. Echocardiographic findings in PH mice with SMC-specific gain or loss of p53 function**

(A) Quantitative data for immunofluorescence study analyzing the number of p53-positive vascular smooth muscle cells (VSMCs) in SMC-specific gain of p53 function (Myh11-Cre/ERT2; *Mdm4*^fl/fl^ (SMC-Mdm4KO)) or loss of p53 function (Myh11-Cre/ERT2; *Trp53*^fl/fl^ (SMC-p53KO)) models (n=4,4,4). (B–D) Echocardiographic findings in PH mice with SMC-specific gain of p53 function (Myh11-Cre/ERT2; *Mdm4*^fl/fl^ (SMC-Mdm4KO)) or loss of p53 function (Myh11-Cre/ERT2; *Trp53*^fl/fl^ (SMC-p53KO)). (B) Fractional shortening (FS) (n=9,4,11), (C) left ventricular systolic dimension (LVDs) (n=9,4,11), and (D) heart rate (n=9,4,11) in the indicated mice. Data represent the mean ± s.e.m. Analyses were done by 2-way ANOVA, followed by Tukey’s multiple comparison test (A–D).
